# Cardiac remodeling on echocardiogram is related to contrast-associated acute kidney injury after coronary angiography: a cross-section study

**DOI:** 10.3389/fcvm.2023.1173586

**Published:** 2023-11-02

**Authors:** Qingqing Chen, Duanbin Li, Hangpan Jiang, Tianli Hu, Yecheng Tao, Changqing Du, Wenbin Zhang

**Affiliations:** ^1^ Department of Cardiology, Affiliated Zhejiang Hospital, College of Medicine, Zhejiang University, Hangzhou, China; ^2^ Department of Cardiology, Sir Run Run Shaw Hospital, College of Medicine, Zhejiang University, Hangzhou, China; ^3^ Key Laboratory of Cardiovascular Intervention and Regenerative Medicine of Zhejiang Province, Hangzhou, China; ^4^ Department of Cardiology, The Fourth Affiliated Hospital, College of Medicine, Zhejiang University, Yiwu, China

**Keywords:** echocardiography, cardiac remodeling, contrast associated acute kidney injury, coronary angiography, percutaneous coronary intervention

## Abstract

**Background:**

Cardiac dysfunction is a well-established risk factor for contrast-associated acute kidney injury (CA-AKI). Nevertheless, the relationship between cardiac remodeling, as assessed by echocardiography, and CA-AKI remains uncertain.

**Method:**

A total of 3,241 patients undergoing coronary angiography (CAG) with/without percutaneous coronary intervention (PCI) were enrolled in this retrospective study. Collected echocardiographic parameters were normalized by body surface area (BSA) and divided according to quartile, including the left ventricular internal end-diastolic diameter index (LVIDDI), left ventricular internal end-systolic diameter index (LVIDSI), and left ventricular mass index (LVMI). Logistic regression analysis was conducted to ascertain the association between structural parameter changes and CA-AKI. Further investigation was performed in different subgroups.

**Results:**

The mean age of the participants was 66.6 years, and 16.3% suffered from CA-AKI. LVIDSI [≥22.9 mm/m^2^: OR = 1.953, 95%CI (1.459 to 2.615), *P* < 0.001], LVIDDI [≥33.2 mm/m^2^: OR = 1.443, 95%CI (1.087 to 1.914), *P* = 0.011], and LVMI [≥141.0 g/m^2^: OR = 1.530, 95%CI (1.146 to 2.044), *P* = 0.004] in quartile were positively associated with CA-AKI risk in general (all *P* for trend <0.05). These associations were consistent when stratified by age, left ventricular ejection fraction, estimated glomerular filtration rate, and N-terminal brain natriuretic peptide (all *P* for interaction >0.05). The presence of eccentric hypertrophy [OR = 1.400, 95%CI (1.093 to 1.793), *P* = 0.008] and the coexistence of hypertrophy and dilation [OR = 1.397, 95%CI (1.091 to 1.789), *P* = 0.008] carried a higher CA-AKI risk.

**Conclusion:**

The presence of cardiac remodeling, assessed by echocardiography, is associated with a higher risk of CA-AKI.

## 1. Introduction

Contrast-associated acute kidney injury (CA-AKI) is a dreadful complication characterized by a rapid deterioration of renal function within 72 h after administrating the iodine contrast medium (1). The incidence of CA-AKI has increased recently following the widespread application of coronary angiography (CAG) and percutaneous coronary intervention (PCI) to diagnose and treat coronary artery disease (CAD) (2).

CA-AKI has been ranked as the third most common cause of nosocomial acute kidney injury in the United States, with a 30% rate among patients undergoing CAG/PCI (1). Patients suffering from CA-AKI tend to confront longer hospitalization but worse long-term prognosis and even permanently damaged renal function (3). Therefore, as for patients receiving CAG/PCI, CA-AKI has been a growing health concern, and targeting patients with a high risk of CA-AKI is imperative for clinicians. Existing research has demonstrated the significant association of impaired organ function with CA-AKI risk, such as cardiac dysfunction (4, 5).

Cardiac dysfunction commonly accompanies cardiac remodeling, which involves both functional and structural changes. Cardiac remodeling occurs in both cardiovascular and non-cardiovascular diseases in compensatory response to the overload (6). More than a compensatory change, cardiac remodeling not only plays a formidable role in the progression of cardiac dysfunction to heart failure but also participates in the functional impairment of the kidney (7). Accumulating evidence demonstrates the remarkable correlation between cardiac remodeling and the development of chronic kidney disease (CKD); in turn, impaired renal function is more susceptible to developing into cardiac remodeling (8, 9). As for patients undergoing operation, hemodynamic perturbation caused by decreased cardiac function can be attributed to postoperative acute kidney injury (10, 11). Nevertheless, the association between the presence of cardiac remodeling and CA-AKI risk after CAG/PCI remains unknown.

Accordingly, the current retrospective research was conducted to figure out the association of cardiac remodeling with CA-AKI risk in a cohort of CAG patients undergoing CAG/PCI. With the help of an echocardiogram, certain structural parameters were measured to evaluate cardiac remodeling (6). Further investigation into the association between different patterns of cardiac remodeling and CA-AKI risk was also conducted.

## 2. Materials and methods

### 2.1. Study design

Conforming to the STROBE (Strengthening the Reporting of Observational Studies in Epidemiology) guidelines, this cross-section study was conducted to explore the association of structural and functional cardiac remodeling on an echocardiogram with the risk of CA-AKI (12). A large cohort of CAD patients was consequently recruited at Sir Run Run Shaw Hospital from March 2018 to February 2022 (Supplementary Figure S1). The inclusion criteria for the enrolled patients were set as follows: (1) had undergone CAG/PCI operation; (2) had available Scr levels on admission and after CAG/PCI with 72 h; (3) had accessible echocardiographic parameters during the hospitalization. The patients with one of the following criteria were excluded: (1) the presence of end-stage chronic kidney disease; (2) had underwent more than one administration of iodine contrast agents (3); being exposed to the usage of nephrotoxic medication during the perioperative phase; (4) suffering from a shock, malignant tumor, and lactation condition; (5) expectant mothers.

This retrospective study has been authorized by the Ethics Committee of the Sir Run Run Shaw Hospital (No. 20201217-36) and performed following the Helsinki statement. Given the nature of a retrospective cross-section study, informed consent could be skipped.

### 2.2. Study endpoint

According to the ESUR (European Society of Urogenital Radiology) guidelines, CA-AKI was set as the primary endpoint, and diagnosing by the Scr level increased by ≥44 μmol/L (0.5 mg/dl), or the proportion increased by ≥25% compared to the level on admission (13).

The secondary outcome was considered the percentage of the Scr elevation, which was evaluated by the difference of the Scr level after the CAG/PCI divided baseline Scr level [the percentage of Scr elevation = (postoperative Scr—baseline Scr)/baseline Scr].

### 2.3. Echocardiographic parameters and criteria

Certain echocardiographic parameters were measured to detect the presence of cardiac structural remodeling during the perioperative period (within 3 days before or after the CAG/PCI). All of the echocardiographic parameters were collected during the hospitalization by the clinical operators with long-term specialized training. The criteria of reference for analysis were from the 2015 American Society of Echocardiography (ASE)/European Association of Cardiovascular Imaging (EACVI) document for chamber quantification (14).

Left ventricular (LV) geometry and ejection fraction (EF) were assessed using two-dimensional (2D) echocardiography. All measurements were standardized to body surface area. LVM was calculated according to the Devereux formula: 0.8 × 1.04 × [(IVSD + LVIDD + LVPWD)^3^– LVIDD^3^] + 0.6 g. Relative wall thickness (RWT) was calculated as 2 × LVPWD divided by LVIDD to depict left ventricular (LV) geometry.

In the 2015 ASE/EACVI document, abnormal LVMI (>95 g/m^2^ in females and >115 g/m^2^ in males) was applied to assess the abnormal LV geometry, and elevated LVIDDI (>30 mm/m^2^ in males and >31 mm/m^2^ in females) and LVIDSI (>21 mm/m^2^) could reflect the enlarged size of the heart chambers to some extent. Therefore, in the present study, LVMI, LVIDDI, and LVIDSI were measured to demonstrate the alterations in the shape and size of the heart, respectively.

According to guidelines in China, the ventricular internal end-diastolic diameter was commonly used to assess the ventricular size, and consequently, elevated LVIDDI was applied to indicate the dilated left ventricle in this study. Abnormally increased LVMI indicates the presence of left ventricular hypertrophy (LVH). Then, with the help of RWT and LVMI, concentric remodeling was defined as RWT > 0.42 and LVMI ≤ 115 g/m^2^ in males or ≤95 g/m^2^ in females; moreover, concentric LVH was diagnosed by RWT > 0.42 and LVMI > 55 mm in males or >50 mm in females, while RWT was ≤0.42 for eccentric LVH.

### 2.4. Definitions and data collection

Data acquired on admission included demographic data, laboratory tests, previous usage of medications, and past history. Fasting blood samples for various clinical routine biochemistry tests and blood examinations at baseline were drawn from antecubital veins. Anemia was defined as hemoglobin <120 g/L in adult males and hemoglobin <110 g/L in adult females (non-pregnant) at sea level. Hypotension was defined as a systolic blood pressure <90 mmHg or a diastolic blood pressure <60 mmHg during the procedure. Abnormal N-terminal pro-brain natriuretic peptide (NT-proBNP) was defined as NT-proBNP exceeding the upper limit of the normal level. For patients <45 years old, the upper limit of normal was 300 pg/ml; for patients between 45 and 70 years of age, the upper limit of normal was 900 pg/ml; and for patients >70 years old, the upper limit of normal was 1,800 pg/ml (15).

The procedures of CAG/PCI for all patients were performed by physicians with longstanding expertise in interventional therapy and acted in accordance with standard practice (16). Detailed procedural data of CAG/PCI were recorded. All patients received perioperative hydration (specific protocol: intravenous infusion of 0.9% saline at a rate of 1 ml/kg/h for 3–4 h preoperatively and 4–6 h postoperatively) (1).

### 2.5. Statistical analysis

Categorical variables were displayed as counts (%) and analyzed by chi-square or Fisher's exact test as appropriate. Continuous data were presented as means ± standard deviations (SD) or median (interquartile range). Comparisons among continuous variables were conducted by the independent Student's *t*-test or non-parametric Mann–Whitney U *t*est in variables with normal distribution or non-normal distribution, respectively.

Between the patients with normal and abnormal echocardiographic parameters (LVIDDI, LVIDSI, and LVMI included), we compared the percentage of Scr elevation through the Mann–Whitney *U* test and the proportion of CA-AKI utilizing the chi-square test. The results of the two analyses were plotted in a violin plot. The associations of echocardiographic structural alterations with the risk of CA-AKI were explored by logistic regression. All of the collected echocardiographic parameters were divided according to quartile. After adjusting for covariables, such as demographic data, laboratory tests, CAG/PCI data, and medication history, multivariable logistic regression was performed, and the restricted cubic spline (RCS) curves were drawn to exhibit the result from the multivariable logistic regression analysis.

The receiver operating characteristics (ROC) curve was drawn to visualize the predictive value of the echocardiographic structural parameters for CA-AKI utilizing the area under curve (AUC). Considering the echocardiographic standard discrepancy between the different sexes, the ROC curve would be plotted for male and female individuals. Given that different individuals had different features, subgroup analysis was performed in various subgroups. Then, we screened the patients with cardiac remodeling that was facilitating changes of the echocardiographic parameters. Further investigation was conducted to explore the association of abnormal LV geometry with CA-AKI development by utilizing multivariable logistic regression. To verify the conclusions drawn from the current study, several sensitivity analyses were performed: first, the status of NT-proBNP was additionally incorporated into multivariable regression models, owing to its relationship with both cardiac function and structure; then, considering that the development of cardiac remodeling has a longstanding progression, multivariable logistic regression was repeated after adjusting the situation of acute cardiac dysfunction, such as myocardial infarction.

A two-tailed *P* value below 0.05 was considered statistically significant. All of the statistical analyses were carried out by SPSS (version 23.0, SPSS Inc., USA) and the R software (version 4.2.0, R core Team 2022).

## 3. Results

### 3.1. Baseline characteristics of CAD patients undergoing CAG/PCI

A total of 3,241 CAD patients undergoing CAG/PCI were incorporated into the study with a mean age of 66.6 ± 11.4 years; their baseline characteristics are displayed in Table 1. Overall, 1,169 (36.0%) patients were female, and 531 (16.3%) patients suffered from CA-AKI following CAG/PCI. As for baseline echocardiographic data, the patients who developed CA-AKI showed higher measurements of echocardiographic structural parameters (LVMI: 120.0 ± 36.9 vs. 132.1 ± 43.3 g/m^2^, LVIDDI: 30.5 ± 4.9 vs. 32.0 ± 5.5 mm/m^2^, LVIDSI: 20.5 ± 5.4 vs. 22.8 ± 6.3 mm/m^2^, all *P* < 0.001) but a lower level of the echocardiographic functional parameter (LVEF: 61.7% ± 11.9 vs. 56.3% ± 13.8%, *P* < 0.001). The presence of LVH was more common in patients suffering from CA-AKI (58.1% vs. 71.1%, *P* < 0.001); meanwhile, the group with CA-AKI exhibited a higher proportion of LV dilation (46.1% vs. 56.6%, *P* < 0.001).

**Table 1:** Baseline characteristics of patients enrolled.

	Overall	Without CA-AKI	With CA-AKI	*P* value
	*N* = 3,247	*N* = 2,716	*N* = 531	
Demographic data				
Female, *n* (%)	1,169 (36.03)	965 (35.53)	205 (38.61)	0.19
Age, years	66.6 ± 11.4	66.4 ± 11.3	67.8 ± 11.7	0.01
BSA, m^2^	1.7 ± 0.2	1.7 ± 0.2	1.6 ± 0.2	<0.01
Smoke (%)	524 (16.14)	448 (16.49)	76 (14.31)	0.24
Drink (%)	481 (14.81)	415 (15.28)	66 (12.43)	0.1
Diabetes (%)	772 (23.78)	621 (22.86)	151 (28.44)	0.01
Hypertension (%)	1,943 (59.84)	1,613 (59.39)	330 (62.15)	0.26
Anemia	582 (17.9)	463 (17.0)	119 (22.4)	0.004
Echocardiographic parameters				
EF, %	60.8 ± 12.4	61.7 ± 11.9	56.3 ± 13.8	<0.01
LVMI, g/m^2^	122.0 ± 38.2	120.0 ± 36.9	132.1 ± 43.3	<0.01
LVIDDI, mm/m^2^	30.8 ± 5.1	30.5 ± 4.9	32.0 ± 5.5	<0.01
LVIDSI, mm/m^2^	20.9 ± 5.6	20.5 ± 5.4	22.8 ± 6.3	<0.01
RWT	0.4 ± 0.1	0.4 ± 0.1	0.4 ± 0.1	0.36
Hypertrophy, *n* (%)	1,957 (60.27)	1,579 (58.14)	378 (71.19)	<0.01
Dilation, *n* (%)	1,555 (47.89)	1,254 (46.17)	301 (56.69)	
Laboratory examination				
CRP, mg/L	1.9 [0.8,5.9]	1.7 [0.8,5.1]	3.5 [1.2,11.2]	<0.01
Proportion of Scr elevation, %	5.5 [−3.2, 17.4]	2.5 [−4.6, 10.6]	39.1 [30.5, 59.6]	<0.01
eGFR, ml/min/1.73 m^2^	93.9 [75.1,113.2]	93.9 [76.2,112.1]	94.1 [69.2,120.1]	0.59
NTproBNP, μg/ml	452.0 [119.0,1427.5]	358.0 [102.0,1108.5]	1475.0 [401.5,3440.0]	<0.01
HbA1c, %	6.4 ± 1.3	6.4 ± 1.3	6.5 ± 1.4	0.03
PCI/CAG Data				
CTO (%)	244 (7.51)	189 (6.96)	55 (10.36)	0.01
Procedure, *n* (%)				0.01
Without PCI	1,935 (59.59)	1,639 (60.35)	296 (55.74)	
Single-vessel PCI	511 (15.74)	434 (15.98)	77 (14.50)	
Multiple-vessel PCI	801 (24.67)	643 (23.67)	158 (29.76)	
Hypotension	439 (13.5)	361 (13.3)	78 (14.7)	0.405
Type of contrast				0.15
Ioversol	544 (16.8)	462 (17.0)	82 (15.4)	
Iohexol	859 (26.5)	735 (27.1)	124 (23.4)	
Iodixanol	1,626 (50.1)	1,337 (49.2)	289 (54.4)	
Iopamidol	218 (6.7)	182 (6.7)	36 (6.8)	
Medication (%)				
Statin	2,693 (82.94)	2,274 (83.73)	419 (78.91)	0.01
ACEI or ARB	1,334 (41.08)	1,117 (41.13)	217 (40.87)	0.95
CCB	938 (28.89)	792 (29.16)	146 (27.50)	0.47

Data are presented as number (percentage) or median (Q1, Q3) for categorical variables and continuous variables. CA-AKI, contrast-associated acute kidney injury; BSA, body surface area; LVEF, left ventricular ejection fraction; LVMI, left ventricular mass index; LVIDDI, left ventricular internal diameters at end-diastole index; LVIDSI, left ventricular internal diameters at end-systole index; RWT, relative wall thickness; NT proBNP, N-terminal pro-brain natriuretic peptide; SCr, serum creatine; CRP, C-reactive protein; eGFR, estimated glomerular filtration rate; CAG, coronary angiography; PCI, percutaneous coronary intervention; CTO, chronic total occlusion; ACEI, angiotensin-converting enzyme inhibitor; ARB, angiotensin receptor blocker; CCB, calcium channel blocker.

Moreover, compared with the patients without CA-AKI, patients in the CA-AKI group had worse renal function (Scr increased proportion: 2.50% [−4.60 to 10.60] vs. 39.10% [30.50 to 59.60], *P* < 0.001), worse cardiac status (NT-proBNP: 358.0 [102.0 to 1108.5] vs. 1475.0 [401.5 to 3440.0] μg/ml, *P* < 0.001), higher inflammatory level (CRP: 1.70 mg/L [0.80 to 5.10] vs. 3.50 mg/L [1.20 to 11.20], *P* < 0.001), higher prevalence of diabetes (22.8% vs. 28.4%, *P *= 0.01), and less frequent usage of statin (83.7% vs. 78.9%, *P *= 0.01).

### 3.2. The associations of echocardiographic parameters with the percentage of Scr elevation and the proportion of CA-AKI

**Figure 1:**
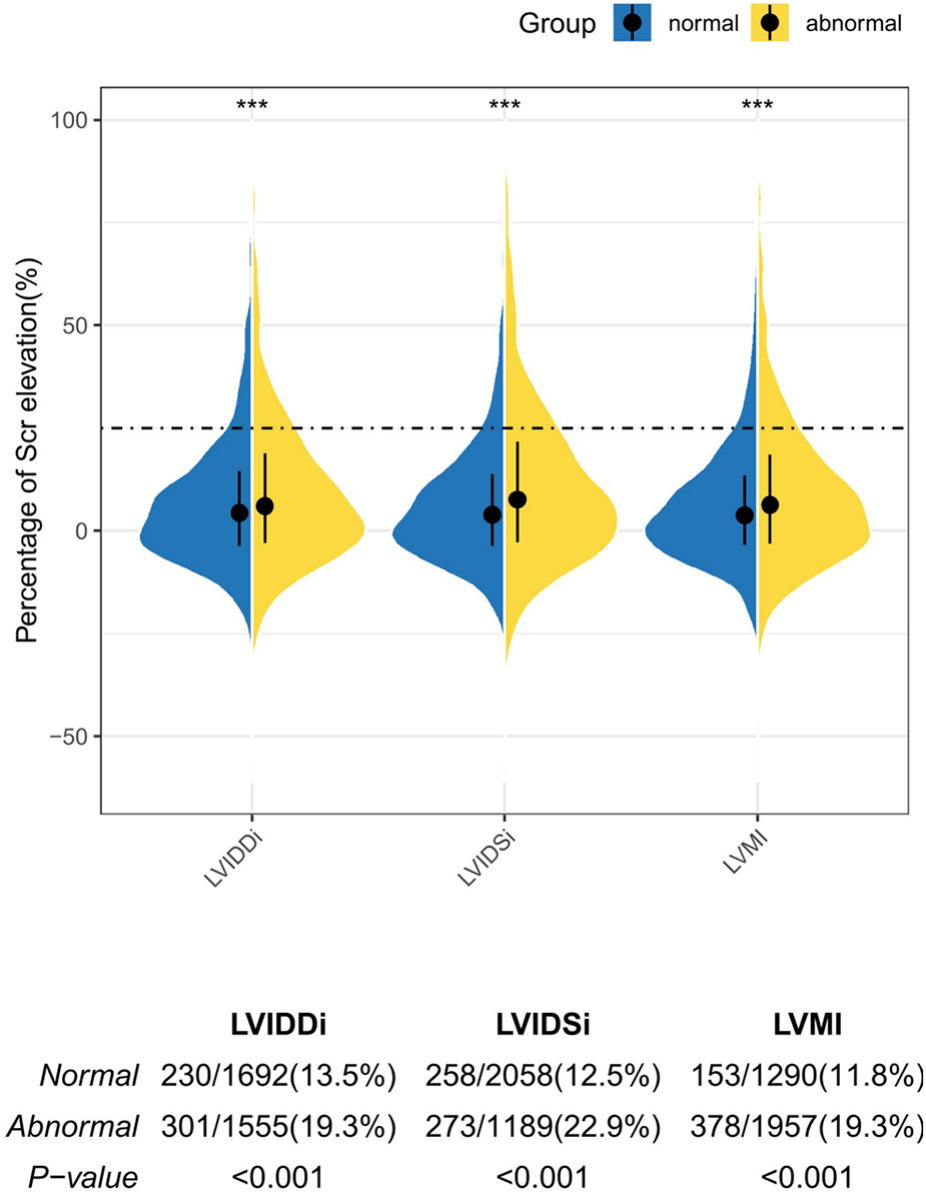
The violin plot depicting the percentage of the Scr elevation in the patients with normal and abnormal echocardiographic parameters. The dashed line represents the diagnostic threshold of CA-AKI. The upper and bottom sides of the boxes indicate Q3 and Q1 separately, with cross-lines presenting the medians. The bilateral curves in different subgroups describe the distribution of the data. Comparisons were performed by the Mann–Whitney *U* test for the difference of the Scr elevation between the normal and abnormal groups and the chi-square test for the difference of the CA-AKI proportion. Asterisks indicate the significance levels: (***) *P* < 0.001.

A violin plot was drawn to uncover the difference in Scr elevation percentage between the groups with abnormal and normal echocardiographic parameters (Figure 1). The abnormal higher structural parameters were not only correlated with a higher percentage of Scr elevation (LVMI: 3.8% vs*.* 6.5%, LVDDI: 4.75% vs*.* 6.40%, LVDSI: 4.20% vs*.* 8.00%, all *P* < 0.001) but also carried a greater proportion of CA-AKI (19.3%, 22.9%, and 19.3% for abnormal LVIDDI, LVIDSI, and LVMI, respectively; all *P* < 0.001).

### 3.3. The associations of CA-AKI with echocardiographic structural and functional alterations

To investigate the association of echocardiographic structural alterations with CA-AKI risk, logistic regression models were established. Echocardiographic structural parameters, including LVMI, LVIDDI, and LVIDSI, were divided into quartiles and then separately incorporated into a logistic regression analysis.

**Figure 2:**
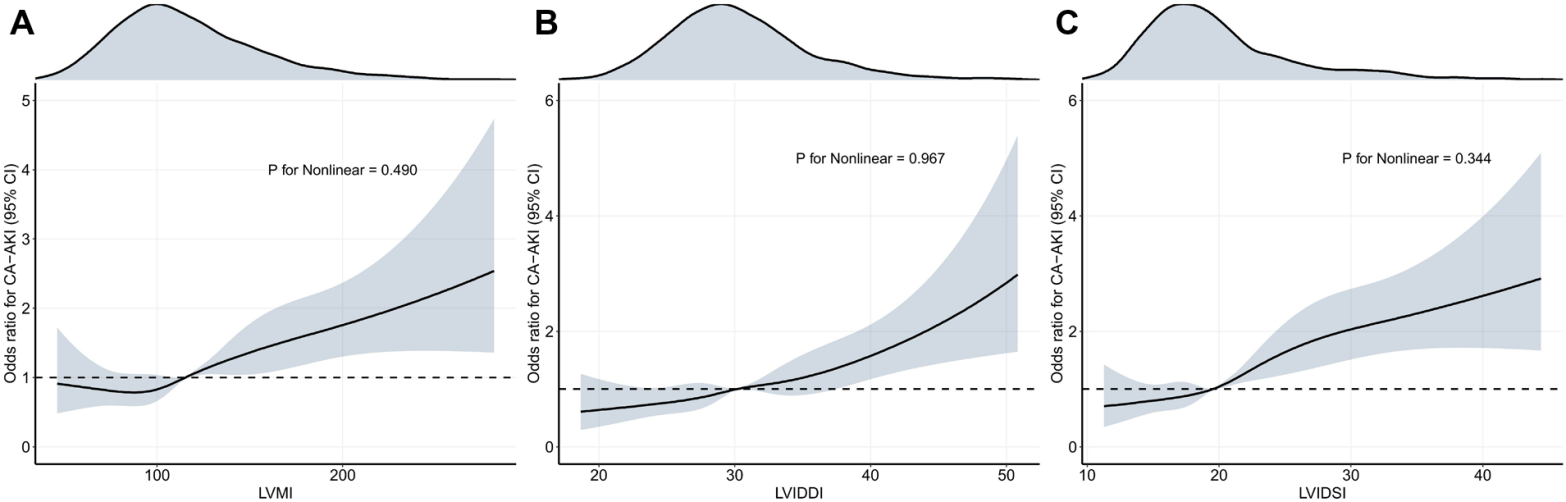
Restricted cubic spline (RCS) analyses for exploring the association of echocardiographic parameters with CA-AKI. The solid black lines show the adjusted odds ratios of different echocardiographic parameters (**A** for LVMI, **B** for LVIDDI, **C** for LVIDSI) for CA-AKI, and the gray ribbon around the line indicates a 95% confidence interval of the curves.

In the univariable logistic regression analysis, the structural parameters were positively associated with CA-AKI risk (Supplementary Table S1). After adjustment for demographic data, laboratory tests, CAG/PCI data, and medication history, such an association between CA-AKI risk and echocardiographic structural parameters was still valid in multivariable logistic regression (Table 2). To be specific, a patient with higher levels of LVMI, LVIDDI, and LVIDSI carried a higher risk of CA-AKI, with a fully adjusted OR of 1.53 [1.146 to 2.044], 1.443 [1.087 to 1.914], and 1.953 [1.459 to 2.615] for the highest quartile compared with the lowest quartile (LVMI: *P* = 0.004; LVIDDI: *P* = 0.011; LVIDSI: *P* < 0.001). Significant linear trends of structural parameters were validated among the consecutive categories (all *P* for trend <0.05), and similar relationship patterns were visualized by the RCS models (Figure 2). The change of CA-AKI risk in different levels appeared as a linear increment, as for LVIDDI, LVIDSI, and LVMI (all *P* for non-linearity >0.05). Such associations between echocardiographic parameters and CA-AKI risk also remained stable in the sensitivity analysis (Supplementary Table S2).

**Table 2:** Multivariable logistic regression analysis of different echocardiographic parameters on CA-AKI.

		OR	CI	*P* value	*P* for trend
LVMI	[Min, 95.0)	1	Ref.		<0.001
	[95.0,115.0)	0.963	(0.709, 1.308)	0.809	
	[115.0,141.0)	1.219	(0.906, 1.641)	0.191	
	[141.0, Max]	1.53	(1.146, 2.044)	0.004	
LVIDDI	[Min, 27.50)	1	Ref.		<0.001
	[27.50,30.00)	0.988	(0.73, 1.339)	0.94	
	[30.00,33.20)	1.218	(0.913, 1.625)	0.179	
	[33.20, Max]	1.443	(1.087, 1.914)	0.011	
LVIDSI	[Min, 17.2)	1	Ref.		<0.001
	[17.2,19.5)	1.245	(0.911, 1.7)	0.169	
	[19.5,22.9)	1.238	(0.914, 1.678)	0.167	
	[22.9,62.4]	1.953	(1.459, 2.615)	<0.001	

The covariates adjusted in multivariable logistic regression included female (yes or no), age (per 10 years), diabetes (yes or no), hypertension (yes or no), CRP (<6 and ≥6 mg/L), eGFR (<30, 30–59, 60–89, ≥90 ml/min × 1.73 m^2^), cTnI (<0.11 and ≥0.11 ng/ml), CAG/PCI procedure (CAG without/with single-vessel/with multiple-vessel PCI), CTO (yes or no), IVUS/OCT/FFR (yes or no), volume of contrast agent (<100 and ≥100 mg), medications (administration of statin) (yes or no), NTproBNP (abnormal or normal), hypotension (no or yes), and anemia (no or yes).

LVMI, left ventricular mass index; LVIDDI, left ventricular internal diameters at end-diastole index; LVIDSI, left ventricular internal diameters at end-systole index.

**Figure 3:**
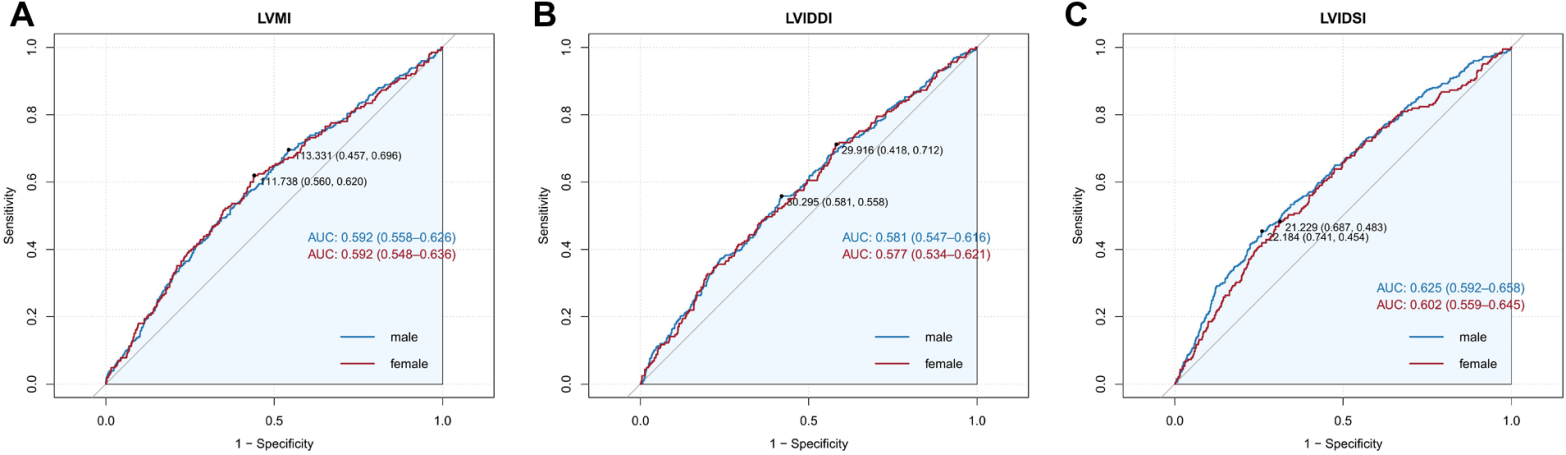
(**A**) The cutoff value of LVMI for predicting CA-AKI was analyzed by the ROC curve. (**B**) The cutoff value of LVIDSI for predicting CA-AKI was analyzed by the ROC curve. (**C**) The cutoff value of LVIDDI for predicting CA-AKI was analyzed by the ROC curve.

Further investigation of the echocardiographic parameters' predictive value was plotted on the ROC curves and exhibited the AUC of the parameters (Figure 3). Considering the structural discrepancy between male and female individuals, the analysis was sex-stratified, and the AUC in different sexes did not exhibit significant differences (LVMI: male vs*.* female = 0.592 vs. 0.592; LVIDDI: male vs. female = 0.581 vs. 0.577; LVIDSI: male vs*.* female = 0.625 vs. 0.602, all *P* > 0.05), which hinted that the predictive value of echocardiographic parameters would not be affected by sex.

### 3.4. The echocardiographic predictor of CA-AKI in different subgroups

**Figure 4:**
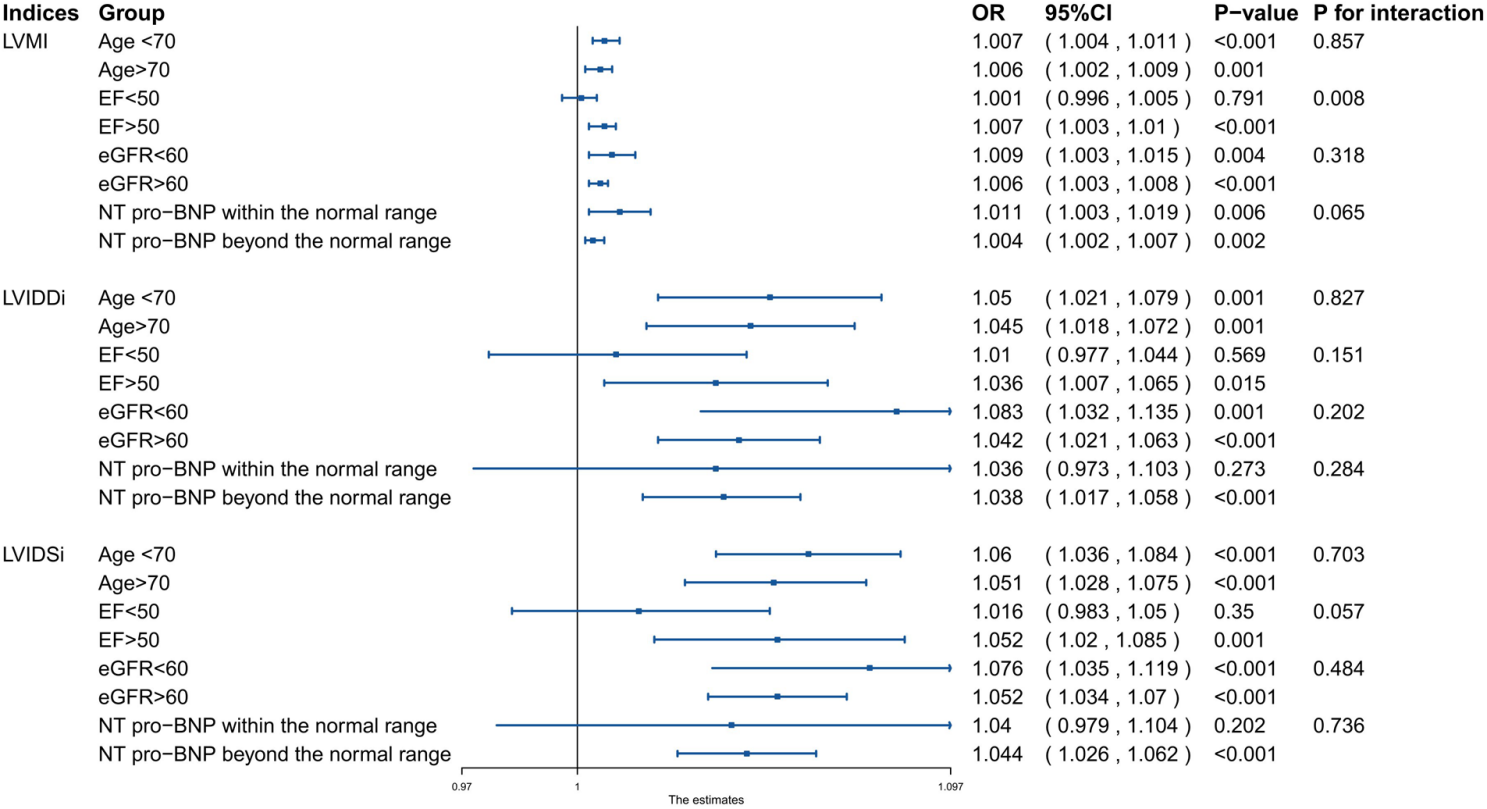
Subgroup analysis of the association between echocardiographic functional and structural parameters and CA-AKI in different individuals.

Considering the distribution of the data among different subgroups, the parameters were taken into subgroup analyses without division (Figure 4). The analysis verified that the positive association between LVIDDI, LVIDSI, and CA-AKI risk was consistent (all *P* for interaction >0.05) when stratified by age (<70 or ≥70 years), left ventricular ejection fraction (<50 or ≥50%), estimated glomerular filtration rate (<60 or ≥60 ml/min/1.73 m^2^), and N-terminal brain natriuretic peptide (normal or abnormal).

### 3.5. The associations between CA-AKI and abnormal left ventricular geometry

In the current study, abnormal LVMI and LVIDDI signified LV hypertrophy and dilation. Compared with the patients without structural remodeling, the two abnormal LV geometries were found to possess a significant relationship with CA-AKI risk in the multivariable logistic analysis (hypertrophy: OR = 1.348, 95%CI [1.086 to 1.673], *P* = 0.007; dilation: OR = 1.234, 95%CI [1.012 to 1.506], *P *= 0.038, in Table 3). By means of RWT and LVMI, four types of LVH were classified. The association between LVH and CA-AKI risk was significant in patients with eccentric LVH [OR = 1.400, 95%CI (1.093 to 1.793), *P* = 0.008]. Then, recruited patients were roughly categorized into three statuses: normal LV geometry, the existence of LVH or dilation, and the coexistence of LV hypertrophy and dilation. Multivariable logistic regression was performed to verify the correlation between abnormal LV geometry and CA-AKI risk, which demonstrated that the coexistence of LV hypertrophy and dilation carried the highest risk of CA-AKI [OR = 1.397, 95%CI (1.091 to 1.789), *P* = 0.008].

**Table 3:** Multivariable logistic regression analysis of echocardiographic abnormal ventricular geometry on CA-AKI.

	OR	95%CI	*P* value
Model 1			
Echocardiographic dilatation	1.234	(1.012, 1.506)	0.038
Model 2			
Echocardiographic hypertrophy	1.348	(1.086, 1.673)	0.007
Model 3			
Normal	1	Ref.	
Echocardiographic dilatation/hypertrophy	1.066	(0.812, 1.398)	0.647
Echocardiographic dilatation and hypertrophy	1.397	(1.091, 1.789)	0.008
Model 4			
Normal	1	Ref.	
Concentric remodeling	0.876	(0.569, 1.351)	0.55
Concentric LVH	1.141	(0.852, 1.53)	0.376
Eccentric LVH	1.4	(1.093, 1.793)	0.008

Echocardiographic LV dilation and hypertrophy were incorporated into Model 1 and Model 2, respectively, and various situations of echocardiographic ventricular geometry were incorporated into Model 3.

Adjusted for female (yes or no), age (per 10 years), diabetes (yes or no), hypertension (yes or no), CRP (<6 and ≥6 mg/L), eGFR (<30, 30–59, 60–89, ≥90 ml/min × 1.73 m^2^), cTnI (<0.11 and ≥0.11 ng/ml), CAG/PCI procedure (CAG without/with single-vessel/with multiple-vessel PCI), CTO (yes or no), IVUS/OCT/FFR (yes or no), volume of contrast agent (<100 and ≥100 mg), medications (administration of statin) (yes or no), NTproBNP (abnormal or normal), hypotension (no or yes), and anemia (no or yes).

Echocardiographic LV dilation was diagnosed by abnormal elevated LVIDDI.

Echocardiographic LVH was evaluated by LV mass index (LVMI), >115 g/m^2^ in males or >95 g/m^2^ in females.

Concentric remodeling was set as relative wall thickness (RWT) > 0.42 and LV mass index (LVMI) ≤ 115 g/m^2^ in males or ≤95 g/m^2^ in females, and concentric LVH was diagnosed by RWT > 0.42 and LVMI > 55 mm in males or >50 mm in females, while RWT was ≤0.42 for eccentric LVH.

LVMI, left ventricular mass index; LVIDDI, left ventricular internal diameters at end-diastole index; LVIDSI, left ventricular internal diameters at end-systole index; LVH, left ventricular hypertrophy.

## 4. Discussion

Among this consequent cohort of CAD patients after CAG/PCI, echocardiographic data were available to indicate cardiac remodeling, and 531 (16.3%) patients suffered from CA-AKI. Patients with higher LVMI, LVIDDI, and LVIDSI carried a higher risk of CA-AKI and abnormal LVMI associated with the CA-AKI risk, especially in patients with preserved LVEF. In other words, the presence of cardiac remodeling, namely, left ventricular hypertrophy and dilation, was significantly associated with the CA-AKI risk. The coexistence of LV hypertrophy and dilation possessed the highest risk of CA-AKI among different remodeling patterns, and subjects with eccentric hypertrophy confronted a higher CA-AKI risk.

The development of cardiac remodeling involves alterations in cardiac size, shape, and function (6). An abnormally increased LVMI could detect the occurrence of echocardiographic hypertrophy. LVIDDI and LVIDSI were recorded to evaluate the size of heart chambers. Both the physiological and pathological ways lead to changes in echocardiographic parameters, but it is well established that cardiac remodeling is associated with numerous pathological conditions, including various cardiovascular diseases and chronic kidney disease, among others (17, 18).

One of the leading culprits for cardiac remodeling is hypertension (6). Under the circumstance of long-term hypertension, diffuse fibrosis has been shown to occur in the primary target organ, resulting in the development of remodeling and deteriorated function (7). LV hypertrophy is a common impairment of the target organ in hypertensive patients, considering a marker of uncontrolled hypertension, which exacerbates the progression of kidney disease (19). Patients with LV hypertrophy are susceptible to progressive deterioration in kidney function and increased requirement of dialysis (20). LV dilation not only occurs in the later period of hypertension but also develops due to cardiac myocyte damage, which leads to renal fibrosis and decreased blood flow (21). A cross-sectional study carried out by Kensuke et al. suggested that reduced eGFR is related to both cardiac hypertrophy and dilation (20). As for postoperative renal function, research performed by Lee et al. has validated the predictive role played by LV remodeling during the occurrence of postoperative AKI within 7 days after non-cardiac surgery through RWT (22). LV dilation detected by LVIDD was found to be a predictive value for CA-AKI in CAD patients by Li et al. (23). In the current study, the relationship between left ventricular remodeling and CA-AKI risk was consistent with the previous results. Moreover, this relationship was consistent among different subgroups, while the predictive values of structural parameters were less affected by sex. However, compared with the study conducted by Kensuke et al., no significant difference in RWT between the patients with or without CA-AKI was detected in the present study, which might be explained by the higher baseline level of LVMI and different operation types among the patients enrolled in this study. Furthermore, probably due to a higher LVMI level, 16.3% of the subjects suffered from CA-AKI in the current study, in which the proportion of CA-AKI patients was higher than in the previous study.

Patients with cardiac remodeling often carry more potential risk factors than others, such as diabetes mellitus, anemia, and renal dysfunction. Similar patterns of relationships were also found in the current study for patients with structural remodeling, with a higher CA-AKI risk compared to patients without remodeling. It is conceived that cardiac structural remodeling often relates to decreased stroke volume and worse diastolic function, later showing more severe deterioration in the impaired kidney function than in the systolic function (24–26). The exact mechanisms underlying the association between cardiac remodeling and postoperative AKI have not yet been elucidated. However, the traditional association of LV remodeling with impaired renal function is suspected from the presence of hypertension, which results in glomerular sclerosis (27). The potential contribution of the undetectable alteration in the regional renal perfusion also cannot be ruled out. Haruyama et al. found a relationship between LVH and the percentage of nephrosclerosis, even in kidney donors with normal eGFR (28). The relationship between LV dilation and subclinical renal impairment in hypertensive patients was uncovered in the work conducted by Ratto et al. (29).

Of note is that not only does the presence of cardiac remodeling increase the CA-AKI risk but also the different patterns of cardiac remodeling show different risks. To be specific, patients with both LV hypertrophy and dilation confront the highest risk. Yamanaka et al. also argued that the combination of LV dilation and hypertrophy has the worst prognosis compared with other structural patterns among patients with heart failure with preserved ejection fraction (30). The CASCADE Study showed that patients with end-stage chronic kidney disease carry the highest incidence of both LV hypertrophy and dilation compared to chronic kidney disease patients at other stages, which implies that cardiac structural remodeling and its progression might be closely related to worse renal function at baseline (31).

There are also certain limitations in this retrospective research. First, the authority of the conclusions is inevitably limited by the nature of retrospective cross-section research. Therefore, large-scale prospective research is required in the future to further confirm the association of cardiac remodeling with CA-AKI risks. Second, there was a shortage of the calculation for LVM. The linear method was likely to overestimate LVH, but as the most widespread echocardiographic method to evaluate LV geometry, its usefulness in a tremendous number of clinical trials has been verified (14). Therefore, the discrepancy caused by the different methods might have a few significant influences on the final result. Last, in the current study, we failed to record other detailed medication history, such as the diuretic, which inevitably caused a modest effect on the conclusion.

In conclusion, the present study established a significant association of cardiac remodeling, detected by echocardiogram, with the risk of CA-AKI among CAD patients receiving CAG/PCI. Abnormal structural echocardiographic parameters were correlated with CA-AKI, showing that patients with abnormal cardiac structure should take timely protective measures, even those with LV hypertrophy but preserved LVEF.

## Data Availability

The raw data supporting the conclusions of this article will be made available by the authors, without undue reservation.
