# Supplementary material for: Cardiac remodeling on echocardiogram is related to contrast-associated acute kidney injury after coronary angiography: a cross-section study
Source: Front Cardiovasc Med. 2023 Nov 2;10:1173586. doi: 10.3389/fcvm.2023.1173586 (PMC10652280; doi:10.3389/fcvm.2023.1173586)
Supplement: Supplementary file 1 [file Datasheet1.docx]

Supplementary Material

**Association of Cardiac Remodeling on Echocardiogram with Contrast-associated Acute Kidney Injury after Coronary Angiography: A cross-section study**

**Qingqing Chen^1†^, Duanbin Li^2,3†^, Hangpan Jiang^4^, Tianli Hu^4^, Yecheng Tao^2^, Changqing Du^1*^, Wenbin Zhang^2,3*^**

^1^Department of Cardiology, Zhejiang Hospital, College of Medicine, Zhejiang University, Hangzhou 310000, Zhejiang, People’s Republic of China.

^2^Department of Cardiology, Sir Run Run Shaw Hospital, College of Medicine, Zhejiang University, No 3 East of Qingchun Road, Hangzhou, Zhejiang, China.

^3^Key Laboratory of Cardiovascular Intervention and Regenerative Medicine of Zhejiang Province, Hangzhou, China.

^4^Department of Cardiology, The Fourth Affiliated Hospital, College of Medicine, Zhejiang University, Yiwu 322000, Zhejiang, People’s Republic of China.

*** Correspondence:** Wenbin Zhang: 3313011@zju.edu.cn

# Supplementary Materials

**Table S1** The univariable and multivariable logistic regression analysis of different echocardiographic parameters on the CA-AKI

**Table S2** The multivariable logistic regression analysis of different echocardiographic parameters on the CA-AKI

**Figure S1** Flow chart of inclusion and exclusion of study population

**Figure S2** Spearman correlation among various parameters related to the heart’s structure and function

# Supplementary Tables and Figures

## Supplementary Tables

# Table S1 The univariable and multivariable logistic regression analysis of different echocardiographic parameters on the CA-AKI

|  |  | | | Model 1 | | | | Model 2 | | | P for trend |
| --- | --- | --- | --- | --- | --- | --- | --- | --- | --- | --- | --- |
|  |  |  |  | OR | | 95%CI | P value | OR | 95%CI | P value |  |
| LVMI | | [Min,95.0) | 1 | | Ref. | |  | 1 | Ref. |  | <0.001 |
|  | | [95.0,115.0) | 1.064 | | (0.792, 1.43) | | 0.679 | 1.036 | (0.767, 1.4) | 0.816 |  |
|  | | [115.0,141.0) | 1.506 | | (1.135, 1.999) | | 0.005 | 1.4 | (1.047, 1.873) | 0.023 |  |
|  | | [141.0,Max] | 2.164 | | (1.655, 2.829) | | <0.001 | 1.92 | (1.451, 2.542) | <0.001 |  |
| LVIDDI | | [Min,27.5) | 1 | | Ref. | |  | 1 | Ref. |  | <0.001 |
|  | | [27.5,30.0) | 1.057 | | (0.788, 1.418) | | 0.711 | 1.037 | (0.769, 1.398) | 0.813 |  |
|  | | [30.0,33.2) | 1.363 | | (1.034, 1.795) | | 0.028 | 1.31 | (0.987, 1.738) | 0.062 |  |
|  | | [33.2,Max] | 2.007 | | (1.539, 2.617) | | <0.001 | 1.773 | (1.346, 2.337) | <0.001 |  |
| LVIDSI | | [Min,17.2) | 1 | | Ref. | |  | 1 | Ref. |  | <0.001 |
|  | | [17.2,19.5) | 1.279 | | (0.944, 1.733) | | 0.112 | 1.255 | (0.923, 1.707) | 0.148 |  |
|  | | [19.5,22.9) | 1.618 | | (1.209, 2.167) | | 0.001 | 1.414 | (1.05, 1.905) | 0.023 |  |
|  | | [22.9,Max] | 2.885 | | (2.193, 3.796) | | <0.001 | 2.472 | (1.863, 3.281) | <0.001 |  |

Model 1 was adjusted for none.

Model 2 was adjusted for included female (yes or no), age (per 10 years), diabetes (yes or no), hypertension (yes or no), CRP (<6 and ≥6mg/L), eGFR (<30, 30-59, 60-89, ≥90ml/min×1.73m2), cTnI (<0.11 and ≥0.11ng/ml).

# Table S2 The multivariable logistic regression analysis of different echocardiographic parameters on the CA-AKI

|  |  | | OR | | CI | | | P | | P for trend |  |
| --- | --- | --- | --- | --- | --- | --- | --- | --- | --- | --- | --- |
| **Model 1** |  | |  | |  | | |  | |  |  |
| LVMI | [Min,95.0) | | 1 | | Ref. | | |  | | <0.001 |  |
|  | [95.0,115.0) | | 0.964 | | ( 0.71 , 1.308 ) | | 0.813 | | |  |  |
|  | [115.0,141.0) | | 1.221 | | ( 0.908 , 1.643 ) | | 0.187 | | |  |  |
|  | [141.0,Max] | | 1.55 | | ( 1.161 , 2.068 ) | | 0.003 | | |  |  |
| LVIDDI | [Min,27.50) | | 1 | | Ref. | |  | | | 0.002 |  |
|  | [27.50,30.00) | | 0.997 | | ( 0.737 , 1.35 ) | | 0.986 | | |  |  |
|  | [30.00,33.20) | | 1.215 | | ( 0.912 , 1.62 ) | | 0.184 | | |  |  |
|  | [33.20,Max] | | 1.463 | | ( 1.104 , 1.939 ) | | 0.008 | | |  |  |
| LVIDSI | [Min,17.2) | | 1 | | Ref. | |  | | | <0.001 |  |
|  | [17.2,19.5) | | 1.233 | | ( 0.903 , 1.683 ) | | 0.187 | | |  |  |
|  | [19.5,22.9) | | 1.248 | | ( 0.922 , 1.69 ) | | 0.152 | | |  |  |
|  | [22.9,Max] | | 1.964 | | ( 1.468 , 2.628 ) | | <0.001 | | |  |  |
| **Model 2** |  | |  | |  | |  | | |  |  |
| LVMI | | [Min,95.0) | | 1 | | Ref. |  | | <0.001 | | |
|  | | [95.0,115.0) | | 1.014 | | ( 0.749 , 1.374 ) | 0.928 | |  | | |
|  | | [115.0,141.0) | | 1.372 | | ( 1.023 , 1.84 ) | 0.035 | |  | | |
|  | | [141.0,Max] | | 1.874 | | ( 1.41 , 2.489 ) | <0.001 | |  | | |
| LVIDDI | | [Min,27.50) | | 1 | | Ref. |  | | <0.001 | | |
|  | | [27.50,30.00) | | 1.028 | | ( 0.759 , 1.391 ) | 0.859 | |  | | |
|  | | [30.00,33.20) | | 1.307 | | ( 0.981 , 1.741 ) | 0.068 | |  | | |
|  | | [33.20,Max] | | 1.776 | | ( 1.342 , 2.351 ) | <0.001 | |  | | |
| LVIDSI | | [Min,17.2) | | 1 | | Ref. |  | | <0.001 | | |
|  | | [17.2,19.5) | | 1.239 | | ( 0.909 , 1.689 ) | 0.175 | |  | | |
|  | | [19.5,22.9) | | 1.354 | | ( 1.002 , 1.829 ) | 0.048 | |  | | |
|  | | [22.9,Max] | | 2.352 | | ( 1.767 , 3.131 ) | <0.001 | |  | | |

Adjusted for female (yes or no), age (per 10 years), diabetes (yes or no), hypertension (yes or no), CRP (<6 and ≥6mg/L), eGFR (<30, 30-59, 60-89, ≥90ml/min×1.73m^2^), cTnI (<0.11 and ≥0.11ng/ml), CAG/PCI procedure (CAG without/with single-vessel/with multiple-vessel PCI), CTO (yes or no), IVUS/OCT/FFR (yes or no), volume of contrast agent (<100 and ≥100mg), and medications (administration of statin) (yes or no).

Model 1 was adjusted for the status of NT proBNP (normal or abnormal) additionally, while Model 2 was adjusted by the occurrence of acute myocardial infarction (yes or no).

## Supplementary Figures

**Figure S1** Flow chart of inclusion and exclusion of study population


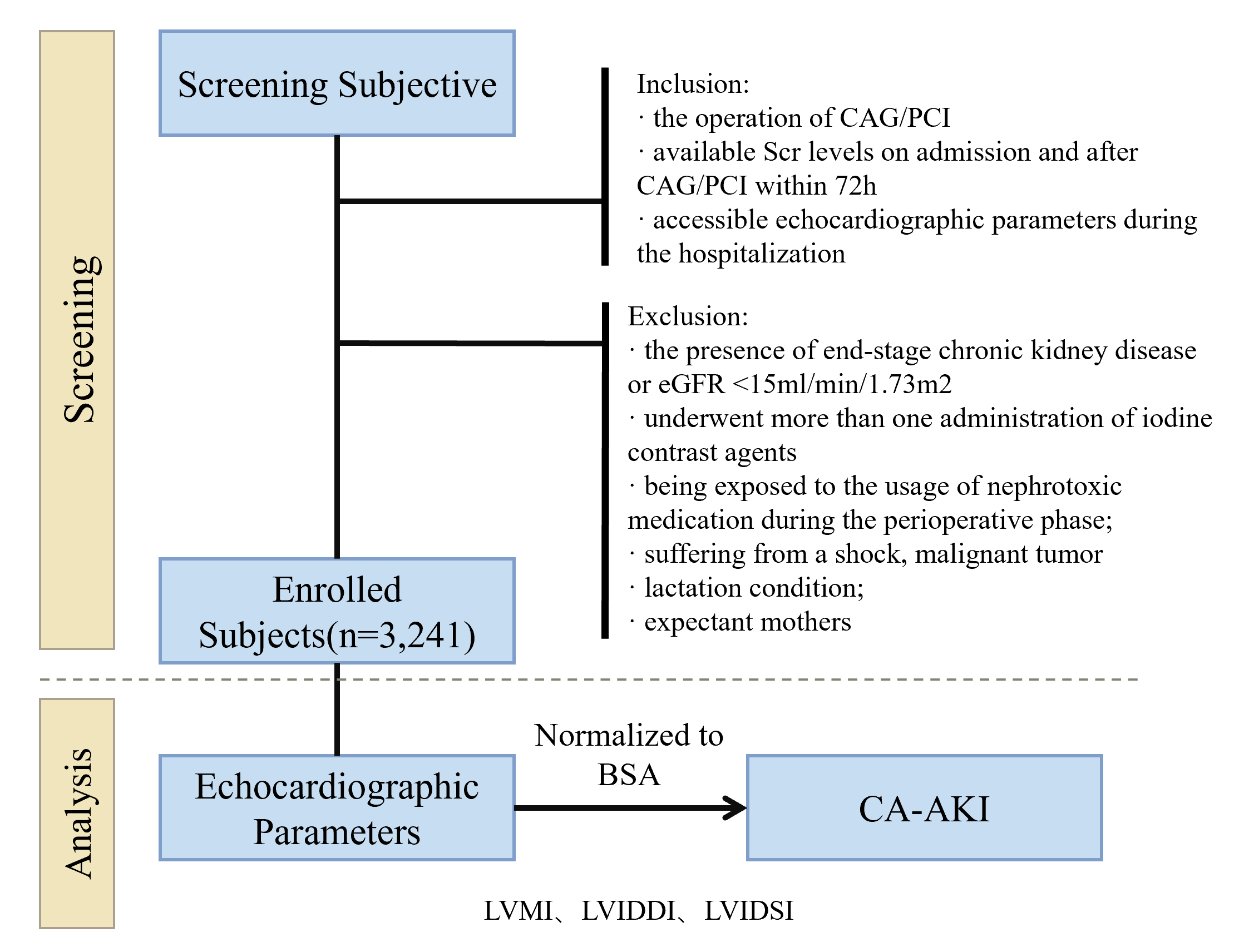


CAG, coronary angioplasty; PCI, percutaneous coronary intervention; CA-AKI, contrast-associated acute kidney injury; LVMI, left ventricular mass index; LVIDDI, left ventricular internal diameters at end-diastole index; LVIDSI, left ventricular internal diameters at end-systole index;

**Figure S2** Spearman correlation among various parameters related to the heart’s structure and function


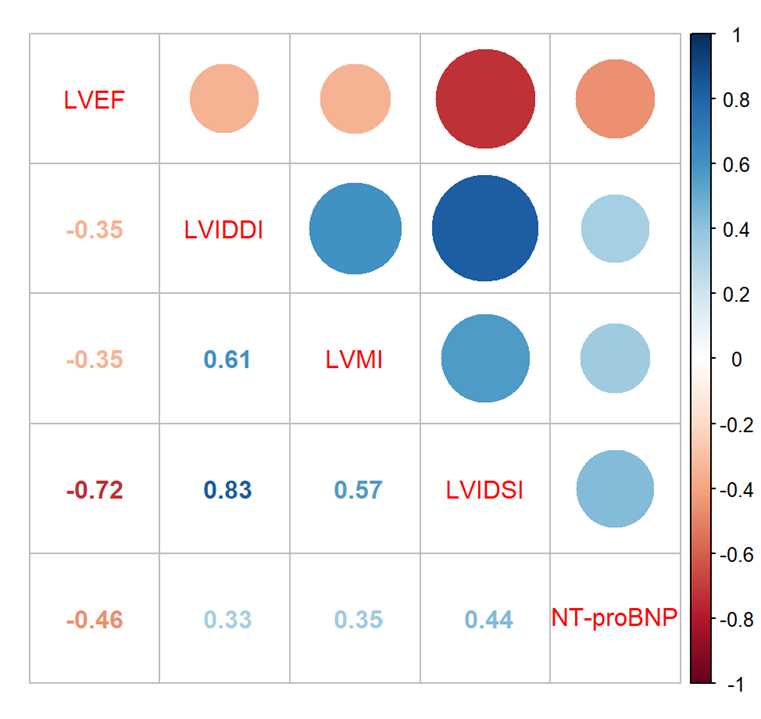


LVMI, left ventricular mass index; LVIDDI, left ventricular internal diameters at end-diastole index; LVIDSI, left ventricular internal diameters at end-systole index; NT proBNP, N-terminal pro-brain natriuretic peptide
